# Physiology of Inflammation and Regeneration in the Organic Tissues

**Published:** 1843-10

**Authors:** 


### ART. XX.

*Physiologie der Entzündung und Regeneration in den Organischen Geweben.* Von Dr. HERMANN KLENCKE.—*Leipzig*, 1842. 8vo, pp. 230.

*Physiology of Inflammation and Regeneration in the Organic Tissues.* By Dr. HERMANN KLENCKE.—*Leipzig*, 1842.

WE have already, more than once, had occasion to speak briefly of some of the many works of Dr. Klencke. They have all been distinguished by the same character of vague idealism as is predominant in this; but, unlike his former publications, this contains a few new facts and experiments, and therefore merits a more lengthened notice than they did. Passing over the first 120 pages, we shall come at once to the *facts*, of which our readers may perhaps make a better use than the doctor himself.

1. *On the regeneration of nerves.* In several experiments, of which four are detailed, Dr. Klencke proved, both by the restoration of function, and by microscopic examination, the reproduction of nervous filaments in the frog; and he says he traced their gradual development through the same series of forms as those through which they pass in original construction. He found also that after division of the pneumogastric nerves of kittens, sensation gradually returned in the parts supplied by them; in these, moreover, he traced the gradual development of the reproduced nervous filaments, and, contrary to the statement of Haighton who assigned six weeks as the period sufficient for restoration, found that reunion of the divided vagus-nerve required sixteen weeks for its completion. In one case he divided the spinal marrow of a dog, and fourteen weeks afterwards the animal could move its hind-legs a little; it twitched its head when they were pricked, and licked its hind feet as if it had sensation in them. After death there appeared to be some restoration of the divided cord; there was a distinctly fibrous bond of union between its two portions, which were chiefly connected by a substance containing strongly developed cells, forming a swelling like a ganglion. In another case, after division of the ischiatic nerve of a kitten, he believes that a genuine ganglion was formed upon the divided part; for, four months after the operation, the limb twitched whenever it was pricked or otherwise stimulated, though the animal had no voluntary power over it; and this twitching continued after all the lumbar nerves had been divided. On examination immediately after death, a round swelling four lines in diameter was found at the part where the nerve had been divided.

"It was covered by a vascular neurilema, which gave the swelling a uniformly glistening, white and not striated appearance. It was now divided longitudinally and examined with a 250 times magnifying power. We found genuine cells such as are found in ganglia, and which here invested fibres which passed without interruption through the swelling near its axis. These fibres exhibited sharp curves, and even knot-like elevations which might be regarded as the cicatrices of the primitive fibres....We discerned a white and a gray substance; the latter consisting of genuine cell matter, the former of fibres. A very compact cellular tissue sent processes from the outer coat into the tumour, and separated in many situations distinct bundles of fibres." (p. 139.)

The author has no doubt this was a genuine ganglion. The observation needs to be repeated by one of a less ideal disposition of mind: though the observations of Arnemann on the restoration of the brain, and two excellent cases in which the author found a complete restoration of both cortical and medullary substance in two soldiers who had lived long after *hernia cerebri*, with great loss of substance, prove that such an event as the production of a ganglion after an injury is far from impossible.

II. *Regeneration of bones, cartilages, and fibrous tissues.* In proof that losses of the wall of a bone may be repaired by growth from the medullary tissue, Klencke describes experiments in which he cut out wedges from the walls of bones and found, after a time, that the spaces were filled up more or less completely by growths of bone from the medullary tissue which was exposed by the removal of the wedges. He describes also two cases of more extensive reproduction after necrosis of part of the wall of a long bone, but they are not such convincing cases as he imagines, for it is not possible to say how deeply the necrosis extended in either case, nor therefore, whether the new wall grew from the surface of the medullary tissue, or from the remaining internal layers of the old wall.

In *internal necrosis* he concludes justly that "the outer layer of the cortical substance which remains healthy, can almost always become the basis for the reproduction of the new bone; and this, not by swelling, expansion, and deposition, but "by actual *catamorphosis* and new formation;" and he adduces several experiments in proof of this, some of which are satisfactory. At the same time he admits that this layer of bone is not essential to the reproduction; and he adds a few to the facts already known, which prove that the periosteum or the surrounding soft parts are capable of forming a new though imperfect bone, where an old one or its shaft, has been wholly destroyed.

Some cases are next adduced in which, after the union of fractures of long bones, the medullary tissue was completely restored; and some in which portions of skull having been removed by the trephine or after necrosis, the holes were nearly filled up by bone. A case is also related in which, in a scrofulous girl, nearly the whole of a scapula was reproduced. The sequestrum comprised all the bone except the acromion, and the surfaces of the edges of the costæ; and it was completely inclosed in new bone presenting, though somewhat roughly, the ordinary form of a scapula.

In the part on *reproduction of cartilage*, there is a probable case of reproduction of part of a costal cartilage in a man wounded five years before death; but on the whole, little addition is made to the few facts which render an occurrence of this kind at all probable. So also, in what is said on the reproduction of muscular and fibrous tissues, and of serous and mucous membranes we find nothing satisfactory. The facts which we have quoted give the work a small degree of interest, but our readers may rest contented that with less labour they have learnt as much from it as we have.

